# Belief in suicide prevention myths and its effect on helping: a nationally representative survey of Australian adults

**DOI:** 10.1186/s12888-020-02715-9

**Published:** 2020-06-15

**Authors:** Angela Nicholas, Thomas Niederkrotenthaler, Nicola Reavley, Jane Pirkis, Anthony Jorm, Matthew J. Spittal

**Affiliations:** 1 Centre for Mental Health, Melbourne School of Population and Global Health, University of Melbourne, Level 4, 207 Bouverie Street, Carlton, Victoria 3053 Australia; 2 Department of Social and Preventative Medicine, Centre for Public Health, Medical University of Vienna, Kinderspitalgasse 15/1 Floor, 1090 Vienna, Austria

**Keywords:** Suicide prevention, Helping, Family, Friends, Peers, Myths, Gatekeeper, Suicide, Intention, Survey

## Abstract

**Background:**

Debunking suicide myths, such as ‘asking someone about suicide could make them start thinking about it’ is a common strategy in suicide prevention education. However, there has been little research investigating the relationship between suicide myths and helping behavior toward people at risk of suicide. We aimed to identify sociodemographic characteristics associated with belief in eight common suicide myths and the associations between beliefs in these myths and helping intentions and behaviors toward a family member or friend in severe distress or at risk of suicide.

**Methods:**

We conducted a random digit dial (mobile and landline) survey of 3002 Australian adults. We asked respondents about their beliefs in suicide myths, intentions to help a person in severe distress or at risk of suicide presented in a vignette, and helping actions taken toward such a person in the last 12 months. We weighted this data to be representative of the Australian population. Regression analyses were undertaken to determine associations between sociodemographic and exposure characteristics and beliefs in suicide myths, and between beliefs in myths and helping intentions and behaviors.

**Results:**

Being male, speaking a language other than English at home and being over 60 years were associated with the strongest beliefs in suicide myths. The strongest and most consistent associations were found between belief in the myth ‘asking someone about suicide could make them start thinking about it’, risk assessment intentions and behaviours and intentions to undertaken actions not recommended for suicide prevention.

**Conclusions:**

Identifying those sociodemographic groups most likely to believe in suicide myths allows targeted intervention for suicide prevention education ‘debunking’ suicide myths. By isolating those myths that are most commonly believed, and their specific effects on helping intentions and behaviors, suicide prevention educators can target these specific myths to have the most effect on helping behavior. Our findings suggest that targeting the myth ‘asking someone about suicide could make them start thinking about it’ may have the greatest effects on helping behavior, and that men, those aged over 60 years and those speaking a language other than English at home could most benefit from myth ‘debunking’.

## Background

In 2017, Australia’s suicide rate was 12.6 per 100,000 persons. This rate has increased from 10.9 suicide deaths per 100,000 persons in 2008 [1]. Although Australian mental health services are currently undergoing significant reforms that may address this issue [2], a high proportion of those who attempt suicide never attend mental health services [3–5]. It is relatively common, however, for them to express their suicidal thoughts in some manner to close family members or friends [4, 6–8]. It is therefore a national imperative that Australians can identify expressions of suicide risk by those close to them, and be willing and able to respond in an appropriate way.

‘Debunking’ suicide myths through education is a common strategy adopted in suicide prevention education aimed at increasing the intentions of community members to assist a person at risk of suicide [9–11]. Adoption of this strategy is based on the assumption that belief in suicide ‘myths’ can have a negative effect on suicide prevention intentions and behaviors [9, 12]. Examples of suicide myths shown to be relatively common among community samples include the belief that people who attempt suicide don’t talk about it [13, 14]; that suicide happens without warning [15–17]; that asking someone about suicide could make them start thinking about it [14, 16, 18]; and that once a person has made up his or her mind about suicide, no one can stop him or her [14, 16]. Such views of suicide may reduce the likelihood of community members intervening with someone who may be at risk because they believe they might make the situation worse or that suicide is not preventable. Beliefs in suicide myths have been shown to be especially common among men [16, 19] and older people [13], and vary widely between cultural groups [20].

Despite the logical assumption that belief in suicide myths affects helping intentions and behavior, empirical testing of this relationship is rare. Evaluation of some gatekeeper training programs for suicide prevention shows that training can reduce belief in suicide myths, while increasing intentions to help or helping behavior, though these evaluations fall short of assessing the link between these constructs [10, 11, 21]. Arendt, Scherr, Niederkrotenthaler, Krallman and Till [17] conducted a randomised controlled trial assessing the effects of exposure to awareness material specifically aimed at debunking suicide myths. This exposure was effective in reducing beliefs in myths and this in turn had a positive effect on participants’ intentions to provide adequate help to individuals at risk of suicide. This finding is, however, restricted purely to helping intentions, and not helping behavior.

We conducted a national survey of Australian adults to assess their confidence, intentions, behaviors and attitudes toward assisting a person they know well who is in severe distress or considering suicide. Findings of this and other studies in our research program [22, 23] were conducted to inform suicide prevention messages for a national suicide prevention campaign aimed at family members and friends of people at risk of suicide (https://www.lifeinmindaustralia.com.au/youcantalk).

In this survey, we assessed respondents’ beliefs in a number of suicide myths, their intentions to help someone in distress or at risk of suicide (presented in a vignette) and their helping actions toward someone at risk of suicide who they had assisted in their own life.

Our analyses aimed to answer three primary research questions:

- Which sociodemographic characteristics are associated with belief in suicide myths?
- Is there an association between endorsement of suicide myths and intentions to help a person in severe distress or at risk of suicide?
- Is there an association between endorsement of suicide myths and types of actions taken to help a person in severe distress or at risk of suicide?

## Methods

We conducted a computer-assisted telephone survey of Australian adults to identify Australian community members’ confidence, attitudes, intentions and behaviors toward helping someone in severe distress or at risk of suicide. We report here on results from those sections of the survey that included questions about attitudes to suicide prevention (belief in ‘suicide prevention myths’), intentions to help a person at risk in severe distress or at risk of suicide and actions taken to help a person in severe distress or at risk of suicide. Findings from other sections of the survey are reported elsewhere [22, 23]. The study was approved by the University of Melbourne Human Research Ethics Committee (Ethics ID: 1648060). Data collection took place from February 14 to March 29, 2017.

### Sampling

To sample 3000 Australian adults, we used a dual frame (mobile and landline) random digit-dial telephone survey method. Landline numbers were from all states and territories of Australia. Six contact attempts were made per telephone number. For landline numbers, interviewers asked to speak to the person associated with each number who satisfied the criteria of being 18 years or over and having the last birthday. The interviewers asked to speak to the person in the household who had the most recent birthday, rather than the person who answered the call, to reduce sampling bias occurring because some members of the household may be more likely than others to answer the landline phone. For mobile phones, interviewers just asked those who answered whether they were 18 years or over. There were 3002 completed surveys. The response rate was 12.8%. This was calculated as the number of completed surveys (*n* = 3002) as a proportion of households where the call was answered and there was an eligible person within that household (i.e., 18 years or over) (*n* = 23, 366). We commissioned Roy Morgan Research Ltd. to conduct the survey. More detail about the sampling method can be found in other publications from our research program [22, 23].

### Survey

#### Suicide myths

We searched the literature to identify existing measures of attitudes to suicide and its prevention and for papers relating to suicide prevention ‘myths’. No measure was found that suited the aims of this study. Rather, we identified several myths commonly believed by lay people [9, 13, 14, 16–18, 24–27] and included seven items in the survey representing these myths: (1) ‘There is a risk that asking someone about suicide will make them start thinking about it’ [14, 16, 18]; (2) ‘There is a risk of making the situation worse if I try to help someone who is thinking about suicide’ [18]; (3) ‘Suicide happens without warning’ [15–17]; (4) ‘Most suicides occur without any sign’ [28]; (5) ‘People who make suicidal threats rarely kill themselves’ [14, 16]; (6) ‘If a person wants to kill themselves, we should not interfere’ [16]; and (7) *‘*Once a person has made up his or her mind about suicide, no one can stop him or her’ [14, 16]. Respondents indicated their agreement with these items on a five-point scale (1. *strongly agree* to 5. *strongly disagree*)*.*

#### Helping intentions

To provide context for the survey questions about intentions to help, we randomly presented respondents with one of six vignettes. These vignettes described a person in severe distress following a series of stressful life events and were based on a vignette used by Jorm et al. in a study of intended mental health first aid behaviors of Australian adults [29]. All vignettes provided a description of a person showing signs of depression and impaired daily functioning. The six versions of the vignette varied by gender (John/Jenny) and directness of communication of suicide risk (distressing life events only, indirect verbal communication of suicide risk, direct verbal communication of suicide risk). The vignettes have been published in full elsewhere [23].

Following presentation of their given vignette, respondents were asked to indicate their likelihood (*1. very unlikely* to 5. *very likely*) of undertaking 13 helping actions. We allocated these helping actions to four subscales: *talking and listening*, *encouraging professional help*, *risk assessment*, and *non-recommended actions.* The non-recommended actions oppose current best practice in suicide prevention [30, 31]. More detail regarding the selection of the 13 actions and allocation to subscales is provided elsewhere [22]. The three items on the *talking and listening* subscale relate to asking the person in distress how they are feeling, listening to their problems without judgement, and asking them ‘how you can help’. The *professional help* subscale items relate to helping the person in distress to make an appointment with a professional, going to the appointment with them, and calling a crisis line. The three *risk assessment* subscale items relate to asking the person in distress if they have been thinking about killing themselves, asking if they have a means to kill themselves*, and asking if they have a plan for suicide*. The *non-recommended actions* subscale items relate to trying to solve the problems of the person in distress, reassuring them you know exactly how they feel, trying to make them understand that suicide is wrong*, and telling them how much it would hurt their friends and family if they were to kill themselves*. The four items indicated with an asterisk are only relevant if John/Jenny has communicated that they are considering suicide. Therefore, when asking respondents how likely they were to undertake these actions, interviewers began each item with ‘If John/Jenny told you he/she was thinking about killing him/herself … ’. Therefore, the directness of suicide communication condition does not apply to these actions.

#### Helping behavior

Interviewers then asked respondents if someone in their family or close circle of friends had experienced distress like John’s/Jenny’s in the last 12 months. If the respondent answered ‘yes’, they were asked if they did anything to support that person. If they did, the interviewer asked if the they had performed the actions included in the *talking and listening* and *encouraging professional help* subscales and the *non-recommended* item related to reassuring the person in distress ‘you know exactly how they feel’ (Yes/No). Interviewers then asked if, during the time respondents were supporting the person in distress, they found out or suspected the person in distress was thinking about suicide. Those who said ‘yes’ were then asked if they performed the actions in the *risk assessment* subscale and the two *non-recommended* items, ‘tell them how much it would hurt their friends and family if they were to kill themselves’; and ‘tried to make them understand that suicide is wrong’.

All respondents were asked if, in the last 12 months, they had considered taking their own life (Yes/No) [32]. All respondents were asked if they had ever had a job that involved providing treatment or services to a person ‘experiencing distress like John’s/Jenny’s?’ (Yes/No), and if they had completed any suicide prevention training (Yes/No). Respondents also answered sociodemographic questions relating to age, gender, language spoken at home, highest level of education, and location of residence.

### Statistical analysis

#### Data preparation

To determine the frequency of beliefs in suicide myths, we re-coded endorsement of each myth (from *strongly agree* to *strongly disagree*) into *agree,* or *do not agree or no view*: *agree* = *agree* and *strongly agree*; *do not agree or no view = neither agree nor disagree, disagree* and *strongly disagree.*

Helping intentions subscale scores were calculated by summing item scores (1 to 5) such that minimum and maximum scores were 3 and 15 for the *talking and listening, encouraging professional help,* and *risk assessment* subscales, and 4 and 20 for *non-recommended actions.* Internal consistency for each of the four intentions subscales is as follows: *talking and listening* (α = 0.64, 3 items)*; encouraging professional help* (α = 0.68, 3 items)*; risk assessment* (α = 0.60, 3 items)*; non-recommended* actions (α = 0.58, 5 items)*.*

Helping behaviors items were also summed to make a score on each of the subscales. The possible range for the *talking and listening, professional help,* and *risk assessment* subscales is therefore 0 to 3, and for the *non-recommended actions* subscale is 0 to 5. Internal consistency for each of the four helping actions subscales was as follows: *talking and listening* (α = 0.53, 3 items); *encouraging professional help* (α = 0.64, 3 items)*; risk assessment* (α = 0.59, 3 items)*; and non-recommended actions* (α = 0.58, 5 items)*.*

#### Data analysis

We generated survey weights and applied these to our sample so that it was representative of the Australian adult population in relation to age, gender, region, and education. Weights were calculated using raking methods [33]. To describe the sample, we first calculated percentages with 95% confidence intervals. To determine if sociodemographic and exposure variables were associated with belief in the suicide prevention myths we conducted logistic regression analysis. We then used linear regression analysis to assess whether belief in suicide prevention myths was associated with helping intentions and behaviors. All logistic and linear regression analyses were adjusted for the following sociodemographic and exposure variables: gender, age, education level, speaking a language other than English at home (LOTE), location, having a family member or friend in distress in the last 12 months, own suicidal ideation in last 12 months, professional experience in suicide prevention, suicide prevention training, and the vignette to which they were exposed. Note that gender of the person in the vignette was shown to have no significant associations with variables of interest, and this was removed from subsequent analyses. All data analyses were carried out in STATA 15.0.

## Results

Table 1 shows the sociodemographic and exposure characteristics of respondents (using weighted data). A substantial minority (39.1, 95% CI 36.9–41.4) stated that a close family member or friend had experienced distress like the person in the vignette, and 84% (95% CI 82.2–9.9) said they had offered that person support. Sixteen percent stated they had considered suicide in the last 12 months (95% CI, 14.3–17.8), and more than half of these received help from a non-professional (52.3, 95% CI 46.3–58.3).

**Table 1:** Descriptive statistics for sociodemographic and exposure characteristics

Characteristic	%	95%CI
Gender		
Female	50.9	48.6, 53.2
Male	49.1	46.8, 51.4
Age, years		
18–30	23.8	21.5, 26.2
31–44	24.5	22.5, 26.7
45–59	24.6	22.9, 26.4
60+	27.1	25.3, 28.9
Speak language other than English at home		
No	91.0	89.5, 92.3
Yes	9.0	7.7, 10.5
Highest Education		
Below Bachelor degree	75.5	73.4, 76.9
Bachelor degree or higher	24.8	23.1, 26.6
Geographic location		
Major city	60.2	57.9, 62.4
Regional, rural or remote	39.8	37.6, 42.1
Vignette		
Life events	34.8	32.6, 37.0
Indirect	31.2	29.1, 33.4
Direct	34.0	31.8, 36.2
Family/friend in distress last 12 months		
Yes	39.1	36.9, 41.4
No	60.9	58.6, 63.1
Offered support to the family member or friend in distress		
Yes	87.6	84.8, 89.9
No	10.7	8.5, 13.3
Own suicidal ideation in last 12 months		
Yes	16.0	14.3, 17.8
No	84.0	82.2, 85.37
Received help from a non-professional when having suicidal thoughts		
Yes	52.3	46.3–58.3
No	47.7	41.7–53.7
Professional experience in suicide prevention		
Yes	19.9	18.2, 21.7
No	80.1	78.3, 81.8
Suicide prevention training		
Yes	15.9	14.3, 17.6
No	84.1	82.4, 85.7

### Endorsement of suicide prevention myths

The most commonly endorsed myths, each endorsed by more than one quarter of respondents, were ‘suicide happens without warning’ (41.5, 95% CI 39.2–43.8); ‘there is a risk that asking someone about suicide will make them start thinking about it’ (30.5%, 28.4–32.6); ‘there is a risk of making the situation worse if I try to help someone who is thinking about suicide’ (29.4%, 27.3–31.5); and ‘most suicides occur without any sign’ (26.7%, 24.7–28.8) (Table 2).

**Table 2:** Percentage frequencies of those who did and did not agree with the suicide prevention myths

	Agree		Do not agree or no view	
Suicide myth	%	95% CI	%	95% CI
Suicide happens without warning.	41.5	39.2, 43.8	58.5	56.2, 60.8
There is a risk that asking someone about suicide will make them start thinking about it.	30.5	28.4, 32.6	69.5	67.4, 71.6
There is a risk of making the situation worse if I try to help someone who is thinking about suicide.	29.4	27.3, 31.5	70.6	68.5, 72.7
Most suicides occur without any sign.	26.7	24.7, 28.8	73.3	71.2, 75.3
People who make suicidal threats rarely kill themselves.	23.6	21.8, 25.6	76.4	74.4, 78.2
Once a person has made up his or her mind about suicide, no one can stop him or her.	11.5	10.2, 12.9	88.5	87.1, 89.8
If someone wants to kill themselves, it is his or her own business and we should not interfere	3.5	2.7, 4.4	96.5	95.6, 97.3

### Associations between sociodemographic and exposure variables and endorsement of myths

Being male, aged over 60 years, or speaking a language other than English at home were each associated with increased odds of endorsing four suicide prevention myths, including that ‘suicide occurs without any sign’ and ‘people who make suicidal threats rarely kill themselves’ (Table 3). Having experienced suicidal thoughts in the last 12 months was strongly associated with endorsement of the myth, ‘If a person wants to kill themselves, we should not interfere’ (OR = 2.80, 95% CI 1.34–4.80). Males, compared with females, had lower odds of endorsing the myth ‘suicide happens without any sign’ (OR = 0.72, 95% CI 0.58–0.89). Those with a Bachelor degree (compared to those with lower education) had lower odds of endorsing the myths that suicide happens without warning or sign, that once the person 'has made up his/her mind no one can stop him/her' and that 'people who make suicidal threats rarely kill themselves'. Those with suicide prevention training (compared with no training) had lower odds of endorsing the myths 'asking about suicide might make them start thinking about it' and 'there is a risk of making the situation worse if I try to help'. Those aged 31 to 44 years had lower odds than those aged 18 to 30 that 'asking about suicide might make them start thinking about it'.

**Table 3:** Multiple logistic regression analysis using sociodemographic and exposure characteristics to predict endorsement of myths

	**Asking about suicide might start them thinking about it**		**Risk making the situation worse if I try to help**		**Suicide happens without warning**		**Suicide occurs without any sign**	
**Predictor**	**OR**	**95% CI**	**OR**	**95% CI**	**OR**	**95% CI**	**OR**	**95% CI**
Gender								
Female	1.0	–	1.0	–	1.0	**–**	1.0	–
Male	1.53**	**1.24, 1.89**	1.37*	**1.11, 1.69**	0.89	0.73, 1.08	0.72*	**0.58, 0.89**
Age, years								
18–30	1.0	–	1.0	–	1.0	**–**	1.0	–
31–44	**0.66**	**0.46, 0.96**	0.92	0.64, 1.31	1.04	0.74, 1.46	0.90	0.61, 1.32
45–59	0.84	0.61, 1.17	0.99	0.71, 1.37	1.24	0.91, 1.69	1.17	0.83, 1.64
60+	1.23	0.89, 1.69	1.42	1.03, 1.96	1.83**	**1.35, 2.48**	1.60*	**1.14, 2.24**
Speak language other than English at home								
No	1.0	–	1.0	–	1.0	**–**	1.0	–
Yes	1.74*	**1.20, 2.52**	0.98	0.66, 1.46	1.46	1.00, 2.12	1.83*	**1.23, 2.73**
Highest Education								
Below Bachelor degree	1.0	–	1.0	–	1.0	**–**	1.0	–
Bachelor degree or higher	0.81	0.65, 1.01	0.97	0.78, 1.22	0.62**	**0.51, 0.77**	0.51**	**0.40, 0.65**
Geographic location								
Major city	1.0	–	1.0	–	1.0	**–**	1.0	–
Regional, rural or remote	0.92	0.74, 1.14	1.00	0.81, 1.23	1.19	0.98, 1.46	1.18	0.95, 1.47
Vignette								
Indirect	1.0	–	1.0	–	1.0	**–**	1.0	–
Direct	1.06	0.83, 1.37	1.32	1.03, 1.69	1.05	0.83, 1.33	1.06	0.82, 1.37
Family/friend in distress last 12 months								
No	1.0	–	1.0	–	1.0	**–**	1.0	–
Yes	0.95	0.76, 1.19	1.02	0.81, 1.28	1.02	0.83, 1.26	1.15	0.92, 1.45
Own suicidal ideation								
No	1.0	–	1.0	–	1.0	**–**	1.0	–
Yes	0.97	0.71, 1.32	1.34	0.99, 1.80	1.23	0.93, 1.62	1.14	0.83, 1.56
Professional experience								
No	1.0	–	1.0	–	1.0	**–**	1.0	–
Yes	1.00	0.74, 1.34	0.97	0.73, 1.30	0.92	0.71, 1.20	0.89	0.66, 1.19
Suicide prevention training								
No	1.0	–	1.0	–	1.0	**–**	1.0	–
Yes	0.67*	**0.47, 0.94**	0.65*	**0.47, 0.91**	0.76	0.56, 1.01	0.84	0.60, 1.17
	**If want to kill themselves, we should not interfere**		**Once made up mind, no one can stop him or her**		**People who make suicidal threats rarely kill themselves**			
**Predictor**	**OR**	**95% CI**	**OR**	**95% CI**	**OR**	**95% CI**		
Gender								
Female	1.0	–	1.0	–	1.0	**–**		
Male	1.67	0.71, 1.90	0.75	0.56, 1.01	1.53**	**1.23, 1.91**		
Age, years								
18–30	1.0	–	1.0	–	1.0	**–**		
31–44	0.84	0.30, 2.31	1.02	0.55, 1.89	1.18	0.78, 1.78		
45–59	0.98	0.44, 2.18	1.85	1.10, 3.11	1.38			
60+	1.99	0.96, 4.16	2.46**	**1.51, 4.01**	2.08**	**1.45, 2.98**		
Speak language other than English at home								
No	1.0	–	1.0	–	1.0	**–**		
Yes	2.80*	**1.27, 6.15**	1.51	0.83, 2.77	1.91*	**1.26, 2.89**		
Highest Education								
Below Bachelor degree	1.0	–	1.0	–	1.0	–		
Bachelor degree or higher	0.74	0.40, 1.37	0.36**	**0.24, 0.53**	**0.73**	**0.57**, **0.94**		
Geographic location								
Major city	1.0	–	1.0	–	1.0	**–**		
Regional, rural or remote	1.03	0.63, 1.70	1.29	0.97, 1.72	1.44*	**1.14, 1.81**		
Vignette								
Indirect	1.0	–	1.0	–	1.0	–		
Direct	1.16	0.61, 2.21	0.94	0.66, 1.34	1.00	0.76, 1.31		
Family/friend in distress last 12 month								
No	1.0	–	1.0	–	1.0	–		
Yes	0.69	0.39, 1.21	1.16	0.86, 1.57	0.93	0.73, 1.18		
Own suicidal ideation								
No	1.0	–	1.0	–	1.0	–		
Yes	2.54*	**1.34, 4.80**	1.23	0.80, 1.89	1.07	0.78, 1.49		
Professional experience								
No	1.0	–	1.0	–	1.0	–		
Yes	0.80	0.40, 1.58	1.31	0.88, 1.95	0.90	0.78, 1.49		
Suicide prevention training								
No	1.0	–	1.0	–	1.0	–		
Yes	1.28	0.61, 2.67	0.75	0.48, 1.16	0.77	0.54, 1.08		

Significant associations are indicated in bold type. *p < 0.05, **p < .001

Significant associations identified in these logistic regression analyses are summarised in Table 4.

**Table 4:** Sociodemographic and exposure variables predicting belief in suicide myths (↓ = lower odds, ↑ higher odds)

Sociodemographic or exposure characteristic	Asking about suicide might start them thinking about it	Risk making the situation worse if I try to help	Suicide happens without warning	Suicide occurs without any sign	If wants to kill themselves, we should not interfere	Once a person has made up mind, no one can stop him/her	People who make suicidal threats rarely kill themselves
Male	**↑**	**↑**		**↓**			**↑**
Aged 60+			**↑**	**↑**		**↑**	**↑**
Speaks language other than English at home	**↑**			**↑**	**↑**		**↑**
Bachelor degree or higher			**↓**	**↓**		**↓**	↓
Regional, rural or remote location							**↑**
Own suicidal ideation					**↑**		
Suicide prevention training	**↓**	**↓**					

### Associations between endorsement of suicide prevention myths and helping intentions and behaviors

Table 5 shows the results of linear regression analyses of the associations between suicide prevention myths and helping intentions and behaviors. Endorsement of the myth, ‘If a person wants to kill themselves, we should not interfere’ had the strongest associations with helping intentions, showing a negative association with both talking and listening (coef. = − 1.13, 95% CI -1.83, − 0.44, *p* < 0.001) and non-recommended intentions (coef. = − 1.51, 95% CI -2.40, − 0.61, *p <* 0.001). It also had a weaker negative association with talking and listening behavior (coef. = − 0.37, 95% CI -0.72, − 0.01, *p* < 0.05). Endorsement of the myth, ‘asking about suicide might start them thinking about it*’* had negative associations with risk assessment intentions (coef. = − 1.30, 95% CI -1.64, − 0.94, *p* < 0.001), risk assessment behaviors (coef. = − 0.47, 95% CI -0.77, − 0.17, *p <* 0.05); and talking and listening intentions (coef. = − 0.18, 95% CI -0.35, − 0.01, *p <* 0.05). ‘Suicide happens without warning’ had a positive association with non-recommended intentions (coef. = 0.52, 95% CI 0.21, 0.82, *p* < 0.001) and behaviors (coef. = 0.48, 95% CI, 0.24–0.73, *p* < 0.001). ‘Suicide happens without any sign’ had a positive association with risk assessment intentions (coef. = 0.47, 95% CI, 0.07–0.86, *p* < 0.001).

**Table 5:** Linear regression analyses of the associations between belief in suicide myths and helping intentions, and between belief in suicide myths and helping behaviors, adjusted for sociodemographic characteristics

	Talk and listen		Professional help		Risk assessment		Non-recommended actions	
Myth	Coefficient	95%CI	Coefficient	95%CI	Coefficient	95%CI	Coefficient	95%CI
**Helping intentions**								
Asking about suicide might start them thinking about it	−0.18*	**− 0.35, − 0.01**	− 0.06	− 0.34, 0.21	−1.30**	**−1.64, − 0.94**	0.19	− 0.10, 0.49
Risk making the situation worse if I try to help	− 0.17*	**− 0.33, − 0.02**	− 0.17	− 0.44, 0.11	−0.00	− 0.35, 0.35	−0.65**	**− 0.96, − 0.34**
Suicide happens without warning	−0.06	− 0.20, 0.09	0.04	− 0.23, 0.30	0.06	− 0.28, 0.41	0.52**	**0.21, 0.82**
Suicide occurs without any sign	0.02	−0.14, 0.18	0.05	−0.26, 0.35	0.47*	**0.07, 0.86**	0.54**	**0.22, 0.87**
If wants to kill themselves, we should not interfere	−1.13**	**−1.83, − 0.44**	−0.83	−1.74, 0.08	− 0.86	− 1.89, 0.17	−1.51**	**−2.40, − 0.61**
Once a person has made up mind, no one can stop him/her	0.06	− 0.17, 0.29	− 0.12	− 0.50, 0.26	0.09	− 0.39, 0.58	0.26	− 0.18, 0.70
People who make suicidal threats rarely kill themselves	− 0.13	− 0.31, 0.05	− 0.28	−0.57, 0.02	− 0.13	−0.51, 0.25	0.32	0.01, 0.63
**Helping behaviors**								
	**Coefficient**	**95%CI**	**Coefficient**	**95%CI**	**Coefficient**	**95%CI**	**Coefficient**	**95%CI**
Asking about suicide might start them thinking about it	−0.05	−0.13, 0.04	0.04	−0.16, 0.24	−0.47*	**− 0.77, − 0.17**	−0.05	− 0.29, 0.20
Risk of making the situation worse if I try to help	0.01	−0.07, 0.08	0.07	−0.13, 0.27	−0.17	− 0.47, 0.13	−0.04	− 0.30, 0.22
Suicide happens without warning	0.05	−0.01, 0.12	0.17	−0.04, 0.38	0.11	−0.17, 0.39	0.48**	**0.24, 0.73**
Suicide occurs without any sign	0.04	−0.03, 0.11	0.06	−0.15, 0.27	0.12	−0.18, 0.42	0.08	−0.18, 0.34
If wants to kill themselves, we should not interfere	−0.37*	**−0.72, − 0.01**	−0.27	− 0.73, 0.20	−0.13	− 0.70, 0.44	−0.07	− 0.82, 0.69
Once a person has made up mind, no one can stop him/her	−0.16	− 0.12, 0.09	−0.11	− 0.41, 0.18	−0.23	− 0.65, 0.20	−0.01	− 0.41, 0.38
People who make suicidal threats rarely kill themselves	−0.12*	**− 0.22, − 0.01**	−0.00	− 0.22, 0.21	−0.06	− 0.35, 0.24	−0.10	− 0.38, 0.19

Significant associations are indicated in bold type. *p < 0.05, **p < .001**.** All analyses adjusted for gender, age, education level, speaking a language other than English at home (LOTE), location, own suicidal ideation in last 12 months, professional experience in suicide prevention, suicide prevention training, and the vignette to which they were exposed. Intentions analyses were also adjusted for having a family member or friend in distress in the last 12 months

Table 6 illustrates the significant relationships identified in regression analyses using endorsement of suicide myths as predictors of helping intentions and behaviors.

**Table 6:** Significant relationships between belief in suicide prevention ‘myths’ and helping intentions and behaviors (↓ = negative association, ↑ positive association)

	Talk and listen		Professional help		Risk assessment		Non-recommended behaviors	
Myth	Intention	Behavior	Intention	Behavior	Intention	Behavior	Intention	Behavior
Asking about suicide might start them thinking about it	**↓**				**↓**	**↓**		
Risk of making the situation worse if I try to help	**↓**						**↓**	
Suicide happens without warning							**↑**	**↑**
Suicide occurs without any sign					**↑**		**↑**	
If wants to kill themselves, we should not interfere	**↓**	**↓**					**↓**	
Once a person has made up mind, no one can stop him/her		**↓**						
People who make suicidal threats rarely kill themselves		**↓**						

## Discussion

We conducted a nationally representative survey of Australian adults to determine the answers to three primary research questions: (1) which sociodemographic characteristics are associated with belief in suicide myths; (2) are beliefs in suicide myths related to people’s intentions to carry out recommended and non-recommended helping actions; and (3) are beliefs in suicide myths associated with actions taken to help someone in severe distress or at risk of suicide.

The most commonly endorsed myths were those that suicide occurs without warning (41.5%) or sign (26.7%); and those related to fears of making the situation worse by asking about suicide (30.5%) and trying to help (29.4%). Being male, aged 60+ and speaking a language other than English at home had the most consistent associations with endorsement of suicide prevention myths. These groups had elevated odds of believing four of the seven myths, including ‘people who make suicidal threats rarely kill themselves’ and ‘suicide occurs without any sign’. Having undertaken suicide prevention training demonstrated positive effects, being associated with reduced odds of believing the two myths about making the situation worse by asking about suicide and trying to help. The two suicide myths having the strongest and most diverse effects on intentions and behaviors were ‘asking about suicide might start them thinking about it*’* and ‘if a person wants to kill themselves, we should not interfere’.

### Belief in suicide prevention myths in Australia

Those suicide prevention myths most commonly endorsed by Australian adults in our study related to a fear of worsening the situation by asking the person at risk directly about suicide or by trying to help and to the unpredictability of suicide. The belief that people who make ‘threats’ about suicide don’t carry them out was also common. These myths persist in almost one quarter or more of of the population. Similar levels of endorsement of these most common suicide prevention myths are reported by Hjelmeland and Loa Knizek [14] in a 2004 study with Norwegian adults and in the 1990 study of adults from the United States by Domino [26]. Given that these studies were conducted more than a decade ago, it would seem the suicide prevention literacy of Australians requires significant development.

This is particularly true of men, older people and those who speak a language other than English at home, who had the greatest odds of believing several suicide prevention myths. Swedish and Austrian studies have similarly found that men more commonly believe several suicide prevention myths regarding the predictability of suicide and that communication of suicidal thoughts means there is no real suicidal intent [16, 19]. Till et al. also recently reported that ‘older age consistently predicted the endorsement of suicide myths’ (p.4) [13]. In our study, the countries of origin of those respondents who spoke a language other than English at home were diverse (e.g., from Mediterranean, South Asian and South East Asian Countries), and we were therefore unable to conduct further evaluations of cultural sub-groups. However, previous research indicates vast cultural differences in beliefs about suicide prevention [20]. It is possible that beliefs in suicide prevention myths reflect a broader lack of mental health literacy, consistent with the findings of a large-scale Australian study, which found that men, those aged over 60 years, and those with a lower level of education have lower levels of general mental health literacy [34]. It is notable that there are numerous suicide prevention programs targeting these population sub-groups (e.g., the Australian *Mates in Construction* and *Mates in Mining* programs aimed at men in male-dominated, high suicide risk professions) and that addressing suicide myths is a common inclusion in such programs. The findings of this study support this approach.

### Effects of endorsement of myths on helping intentions and behaviors

‘Asking about suicide might start them thinking about it*’* was associated with a reduction in both the intention to ask risk assessment questions and of having asked risk assessment questions when supporting a person at risk. This finding illustrates the importance of targeting this myth in suicide prevention education programs, particularly as it was endorsed by almost one-third of the population. In previous studies, we have also found risk assessment intentions and action to be relatively uncommon [22, 23]. Debunking this myth could help to reduce anxiety about causing harm by asking direct questions about suicide and enhance the likelihood of family and friends asking questions of those at risk about suicidal thoughts, plans and means. Debunking of this myth may be particularly important for men and those who speak a language other than English at home. Our findings also show that suicide prevention training is associated with a reduction in beliefs in this myth.

Believing ‘If a person wants to kill themselves, we should not interfere’ was associated with a reduction in talking and listening intentions and behavior, but also with reduced intentions to undertake non-recommended behaviors. While no other associations between this myth and intentions and behaviors were significant, they were all negative, suggesting that people who believe this myth have reduced intentions to help overall, and therefore undertake fewer helping behaviors of any kind. Belief in this myth is relatively rare among Australian adults, though more likely among those who speak a language other than English at home. This may therefore be a useful target for ‘debunking’ among some culturally and linguistically diverse groups to increase feelings of responsibility for suicide prevention and therefore suicide prevention intentions and actions.

There were relatively few other strong associations between beliefs in suicide myths and intentions and behaviors. Contrary to expectation, endorsement of the myth, ‘suicide occurs without any sign’ was associated with increased intentions to ask risk assessment questions. It seems that those who do not expect to observe any overt warning signs of suicide risk are more likely to ask risk assessment questions to better understand the risk status of the person of concern. While community members should be encouraged to continue asking such questions, education regarding the warning signs of suicide risk and indirect forms of suicide communication is clearly needed for a substantial minority of the Australian population.

#### Limitations

We took significant steps to reduce sampling bias, including by weighting our sample to represent the Australian population in terms of age, gender and location; and by making calls for the phone survey to landlines and mobiles and at various times of the day. However, we gained consent for participation by stating that we were conducting a survey regarding ‘helping people in severe distress’. Therefore, we may have oversampled those with higher mental health literacy or with experience of this type of helping, which may have biased our results toward reporting of more positive attitudes, intentions and behaviors. However, if this were the case, we would expect that our estimates of the proportion of Australians who believe in suicide prevention myths would be underestimates, rather than overestimates. Furthermore, in our previous studies, we also demonstrated that a substantial proportion of our sample intended to, or had undertaken, non-recommended actions toward a person at risk of suicide, while neglecting a number of recommended actions [22, 23].

The internal consistency of some of the intentions and helping actions subscales fell below the broadly accepted minimum of α = 0.7. However, it must be considered that these are largely 3- and 4-item scales, and alpha tends to increase with the number of items [35]. Furthermore, a lower alpha value is likely to lead to underestimating, rather than overestimating associations.

We recognise that Indigenous Australians are at high risk of suicide. While we did collect data from some Indigenous Australians, this was a very small sample. Given this very small sample size, we excluded identification as Aboriginal and/or Torres Strait Islander as a predictor in these analyses. Investigation of the beliefs in suicide myths among Indigenous Australians would be a useful investigation, though different recruitment and data collection methods are likely to be required to be culturally appropriate.

Our study was also cross-sectional, with belief in suicide prevention myths, intentions and behaviors being assessed at one timepoint. This cross-sectional design limits our ability to state whether beliefs in suicide prevention myths and helping intentions prospectively predict helping behaviors.

## Conclusions

Suicide prevention education frequently aims to reduce beliefs in suicide prevention myths, often with the intention of increasing the likelihood of participants assisting a person at risk of suicide in desirable ways [9–11]. However, the results of our study challenge some assumptions of this ‘debunking’ approach, suggesting that beliefs in individual suicide prevention myths may have differing effects on individual helping intentions and behaviors or no effect at all. The pattern of associations between beliefs in individual suicide prevention myths and helping intentions and behaviors was inconsistent, and sometimes counter-intuitive, such as the association between believing that suicide happens without sign and greater intentions to ask risk assessment questions.

Our findings suggest that targeting the myth ‘There is a risk that asking someone about suicide might make them starting thinking about it’, particularly among men and those who speak a language other than English at home, may have the greatest effects on helping intentions and behavior. Reducing beliefs in this myth may increase the likelihood of undertaking risk assessment among a substantial minority of the population. The most appropriate study design to test the effects of debunking suicide myths on suicide prevention behaviors would be an intervention study. This would involve an education intervention aimed at correcting beliefs in the target myths and testing whether this had a subsequent effect on helping intentions and behaviors. This method is often undertaken in assessing the effects of suicide prevention gatekeeper training; however, such a study would require a sufficient follow-up period to allow opportunity for real-life helping to have taken place, and this is relatively rare. Furthermore, we recommend testing the effects of reducing beliefs in specific myths on specific intentions and behaviors in order to enhance the evidence base in this area. Such debunking could also take place on a population level, through targeted suicide prevention campaigns. Though the effects of such interventions are more difficult to establish, assessing whether these types of campaigns can reduce beliefs in suicide myths, and the consequences for helping intentions and behaviors would also be invaluable.

## Data Availability

The datasets generated and/or analysed during the current study are not publicly available due limitations of ethical approval but are available from the corresponding author on reasonable request.

## Abbreviations

Coef.: Coefficient
95% CI: 95% Confidence Interval
OR: Odds Ratio
